# Translation and validation of the Hirschsprung and anorectal malformation quality of life (HAQL) questionnaire in a Danish Hirschsprung population

**DOI:** 10.1007/s00383-024-05634-y

**Published:** 2024-02-09

**Authors:** Kristina Gosvig, Signe Steenstrup Jensen, Hannes Sjölander, Nina Højer Hansen, Sören Möller, Niels Qvist, Mark Bremholm Ellebæk

**Affiliations:** 1 Research Unit for Surgery, Odense University Hospital, Odense, Denmark; 2 University of Southern Denmark, Odense, Denmark; 3 Research Unit for Emergency Medicine, Odense University Hospital, Odense, Denmark; 4 OPEN, Open Patient Data Explorative Network, Odense University Hospital, Odense, Denmark; 5 Centre of Excellence in Gastrointestinal Diseases and Malformations in Infancy and Childhood (GAIN), Odense University Hospital, Odense, Denmark

**Keywords:** Hirschsprung’s disease, Quality of life, Questionnaire

## Abstract

**Background:**

Hirschsprung’s disease (HD) may result in an impaired quality of life (QoL) due to bowel problems, postoperative complications and other health-related issues. The Hirschsprung and Anorectal Malformation Quality of Life (HAQL) questionnaire is a disease-specific instrument developed in the Netherlands to measure the QoL in patients with HD and anorectal malformations. The aim of this study was to translate, culturally adapt and validate HAQL in a Danish Hirschsprung population.

**Material and methods:**

Translation and cultural adaptation were performed according to international guidelines. Invitations to participate in the validation were sent to 401 patients operated for HD during the period from 1985 to 2012. A total of 156 patients completed the translated and culturally adapted Danish versions of HAQL and 35 parents of children and adolescents completed the corresponding parent questionnaire. Reliability was evaluated in terms of internal consistency using Cronbach’s *α* and test–retest reliability using Intraclass Correlation Coefficient for the retest step. Known groups comparison was performed with comparison of mild HD (defined as recto-sigmoidal HD) and serious HD (defined as more proximal disease).

**Results:**

The internal consistency of the dimensions was overall satisfactory for adults and adolescents but more problematic for children, where Cronbach’s *α* was less than 0.7 in 60% of the dimensions. For both children and adolescents, the *α*-value was unsatisfactory for social functioning, emotional functioning, and body image. The test–retest reliability was overall good. The known groups comparison was only able to demonstrate a significant difference between mild and severe HD within one dimension.

**Conclusions:**

The translated version of the HAQL questionnaires provides an overall reliable instrument for evaluating disease-specific QoL in a Danish HD population, but it is important to acknowledge the limitations of the questionnaire, especially in children and adolescents.

## Introduction

Hirschsprung’s disease (HD) is a congenital disease of the enteric neural system characterized by aganglionosis due to failure in the migration of the neural crest cells during embryological development. The aganglionosis is confined to the rectosigmoid region in approximately 75% of the cases, while more severe cases involve the whole colon and/or distal small intestine or even the whole gut in very rare cases. The aganglionosis results in fecal impaction proximal to the affected aperistaltic bowel segment and the only effective treatment is surgical resection of the aganglionic bowel segment [1].

For most patients, surgery involves an anastomosis between the remnant intestine and the anal canal, while patients with extensive aganglionosis as well as patients with complications to surgery or a bad functional outcome may end up with a permanent stoma. Most patients are operated within their first year of life.

The postoperative complications and functional outcome in the early postoperative period have been well documented. Some evidence points to an improvement in functional outcome in the years following surgery, but the long-term quality of life (QoL) for patients with HD is not well explored [2].

Generic QoL measures include common aspects of life relevant to the general public without encompassing disease-specific aspects [3]. The Hirschsprung and Anorectal Malformation Quality of Life (HAQL) questionnaire is an instrument developed to measure the disease-specific QoL in adults, adolescents, and children/parents. It was originally developed in Netherlands based on literature reviews, expert statements and patient interviews [4]. It has since been translated into Swedish, Italian, English and French [5–8]).

## Aim of the study

The aim of this study was to translate, culturally adapt and validate the HAQL questionnaire in a Danish HD population.

## Materials and methods

### Original HAQL questionnaire

The original HAQL questionnaire consists of five different questionnaires [4]; three for patients aged 8–11 years (HAQL_8–11_), 12–16 years (HAQL_12–16_) and 17 years and older (HAQL_ADULT_) and two for parents to children aged 6–11 years (HAQL_PARENT 6–11_) and 12–16 years (HAQL_PARENT 12–16_). The number of dimensions and items in each dimension of the original questionnaires can be found in Table 1. The sexual functioning dimension is only included in HAQL_ADULT_. Items related to amorous feelings and fantasies of making love are included in the social functioning dimension in the HAQL_12–16_.

**Table 1:** Dimensions and number of items for each dimension for the original five different HAQL questionnaires

Dimension	Number of items HAQL_ADULT_	Number of items HAQL_12–16_ HAQL_parents 12–16_	Number of items HAQL_8–11_ HAQL_parents 6–11_
Laxative diet	2	2	2
Constipating diet	2	2	2
Presence of diarrhea	2	2	2
Fecal incontinence	8	8	8
Urinary incontinence	4	4	4
Social functioning	3	5	3
Emotional functioning	7	6	6
Body image	2	2	2
Physical symptoms	9	9	9
Sexual functioning	2	–	–

Each item is phrased as a multiple-choice question, where patients or parents are requested to choose one of four answers: Very often, often, occasionally, or never. All items cover a 1-week time frame, except for the sexual functioning items, which cover a 4-week time frame. Responses are linearly transformed on a 0–100 scale with a higher score indicating better QoL. For patients with a stoma the questions regarding toilet habits and defecation are irrelevant, and these patients are asked to skip these items (a total of 22) and instead answer 8 items regarding symptoms related to their stoma.

Scoring of any given dimension can be computed as the sum of the items scored divided by the number of items answered. At least 50% of the items in a dimension must be answered for the score to be valid. In the original HAQL, a total score was calculated as the sum of all dimension scores. The requirement was an available and valid dimension score for all dimensions.

For each item, the impact of any reported symptom was evaluated with the addition of the question “How much did it bother you?”. This additional question was not consistently reported since the question only can be answered if the patient had experienced the symptom. Therefore, it was not included in the validation of the questionnaire and it has not been validated in the other versions of the questionnaire.

### Translation

Principles from existing guidelines for translation and cultural adaptation were implemented in this study [9, 10]. The English and Swedish versions of the HAQL questionnaires were obtained from the developers of the original study [4]. Denmark and Sweden have cultural and linguistic similarities [11] and the translation was therefore performed from the Swedish version.

A forward/backward translation was performed. The forward translation was carried out by two bilingual translators (main language: Danish, second language Swedish) and the translators finally agreed upon a first forward translation. This translation was then translated backwards by another bilingual translator (main language: Swedish, second language: Danish). The forward and backward translations were made available to each translator and each item was reviewed individually by the entire group of translators at a meeting. Issues and discrepancies were addressed to obtain consensus on a final translated version of the questionnaire. The English version of the HAQL questionnaire was used for reference.

### Cultural adaptation

Face and content validity refers to the comprehensibility and relevance of items. These validity concepts were evaluated through patient interviews and an expert committee review. The questionnaires were distributed to two adult patients with HD, two patients aged 12–16 and their parents and two patients aged 8–11 and their parents. They were instructed to read and answer the questionnaires, followed by a group interview where each item was reviewed individually for understanding and relevance. Comments were registered for each element, but no changes were made to the final translated version at the group interview as per recommendations for cultural adaptation.

An expert committee consisting of two pediatric surgeons, a pediatric gastroenterologist and a specialist nurse was assembled, and each item was reviewed individually, including the comments from the group interview. Changes to the final translated version of the questionnaire were made as a result of expert agreement based on the comments of both patients and experts. This resulted in minor linguistic changes to a few items to make them more comprehensible without changing the content of the item. In the final Danish versions, some of the items were merged and some items were added to make the questionnaires more similar (Table 2).

**Table 2:** Merged and included items in the Danish version of the questionnaires

Dimension	Item	Changes and comments from patients and expert committee
HAQL_ADULT_		
Emotional function	How often have you been ashamed because of your condition?	These items were hard to distinguish in Danish. The questions were altered to one question: How often have you been embarrassed because of your condition?
	How often have you been embarrassed because of your condition?	
	How often have you been uncertain because of your condition?	
	How often did you have to leave a work-related situation (ex. a meeting), because you had to go to the toilet?	This item was added. Inspired by a similar question in the HAQL_12–16_ and HAQL_8–11_
	How often have you assumed that people liked you less because of your condition	This item was added. Inspired by a similar question in the HAQL_12–16_ and HAQL_8–11_
Social functioning	How often has your condition prevented you from participating in exercise activities?	This item was added. Inspired by a similar question in the HAQL_12–16_ and HAQL_8–11_
Physical symptoms	How often have you farted without feeling the urge to fart?	This item was added. Inspired by a similar question in the HAQL_12–16_ and HAQL_8–11_
HAQL_12–16_		
Social functioning	How often did you not express emotions like falling in love because of your condition?	These items were hard to understand in Danish and poorly phrased. The questions were altered to one question: How often have you avoided physical contact (like hugs and kisses) because of your condition?
	How often did you not fantasize about being kissed or having sex because of your condition?	
	How often has your condition prevented you from spending the night elsewhere?	This item was added. Inspired by a similar question in the HAQL_ADULT_
Physical symptoms	How often did you have trouble distinguishing between farts and stool?	This item was added. Inspired by a similar question in the HAQL_ADULT_
HAQL_8–11_		
Social functioning	How often has your condition prevented you from spending the night elsewhere?	This item was added. Inspired by a similar question in the HAQL_ADULT_
Physical symptoms	How often did you have trouble distinguishing between farts and stool?	This item was added. Inspired by a similar question in the HAQL_ADULT_

### Study design

This study was approved by The Regional Committee on Health Research Ethics for Southern Denmark (project ID: S-20140071).

All patients registered in the hospital records with an ICD-8 (795139) or ICD-10 code (DQ431) for HD during the years 1985–2012 at Odense University Hospital (one of the two tertiary referral centers for treatment of HD in Denmark) were invited to participate. Participants < 18 years were contacted through their parents. Invitations were sent via the Danish governmental safe electronic message system (www.e-boks.dk). The first invitation was sent out on the 1st of July 2020. We were allowed to send three reminders with 1 week interval. All patients were asked to fill out a consent form. Patients who consented received another message with further instructions and a link to the questionnaire for online completion. For each item a “I do not wish to answer” was added. For non-completed questionnaire, the participant received a reminder 3 months later. Adult patients who completed the questionnaire were asked to participate in the retest step, with the repeated completion of the questionnaire 4 weeks later and with reminders as described above.

The electronic chart of each participant was accessed to determine extension of bowel aganglionosis and whether the patient had a permanent stoma.

### Assessment of validity, reliability and statistical analysis

Face and content validity were evaluated by patient interview and expert committee review as described above. For further evaluation of face and content validity two questions were added at the end of each questionnaire; “Did you find any of the questions were difficult to understand?” and “Is there something else, you think is relevant?”.

Reliability was evaluated in terms of internal consistency and test–retest reliability. Internal consistency evaluates whether the items in each dimension measure the same construct. As an indicator of homogeneity between items Cronbach’s *α* was calculated. An *α* > 0.70 considered as satisfactory [3].

Test–retest reliability evaluates the reproducibility of a questionnaire. This was evaluated using the Intraclass Correlation Coefficient (ICC) to measure the agreement between two sets of HAQL questionnaires obtained from the same patients with 4 weeks interval. An ICC > 0.60 was considered satisfactory [3].

Known groups comparison validates whether an instrument can discriminate between known groups of patients that are expected to score differently based on for example clinical severity of disease. We divided patients into groups based on the anatomic extension of disease, classifying recto-sigmoidal HD as “Mild HD” and HD in the descending colon and more proximally as “Severe HD”. Patients with ultra-short segment HD and patients with unknown disease severity were excluded from the analysis. For the statistical analysis mean scores for each dimension and the overall score in both were calculated and compared using Student’s *t*-test.

The full data set was reviewed by a biostatistician. Statistical analysis was performed in Stata. Data are presented as mean (± SD) unless otherwise indicated. To be able to compare the total scores of the questionnaires across age-groups, the total scores were divided by the number of dimensions.

## Results

**Fig. 1:**
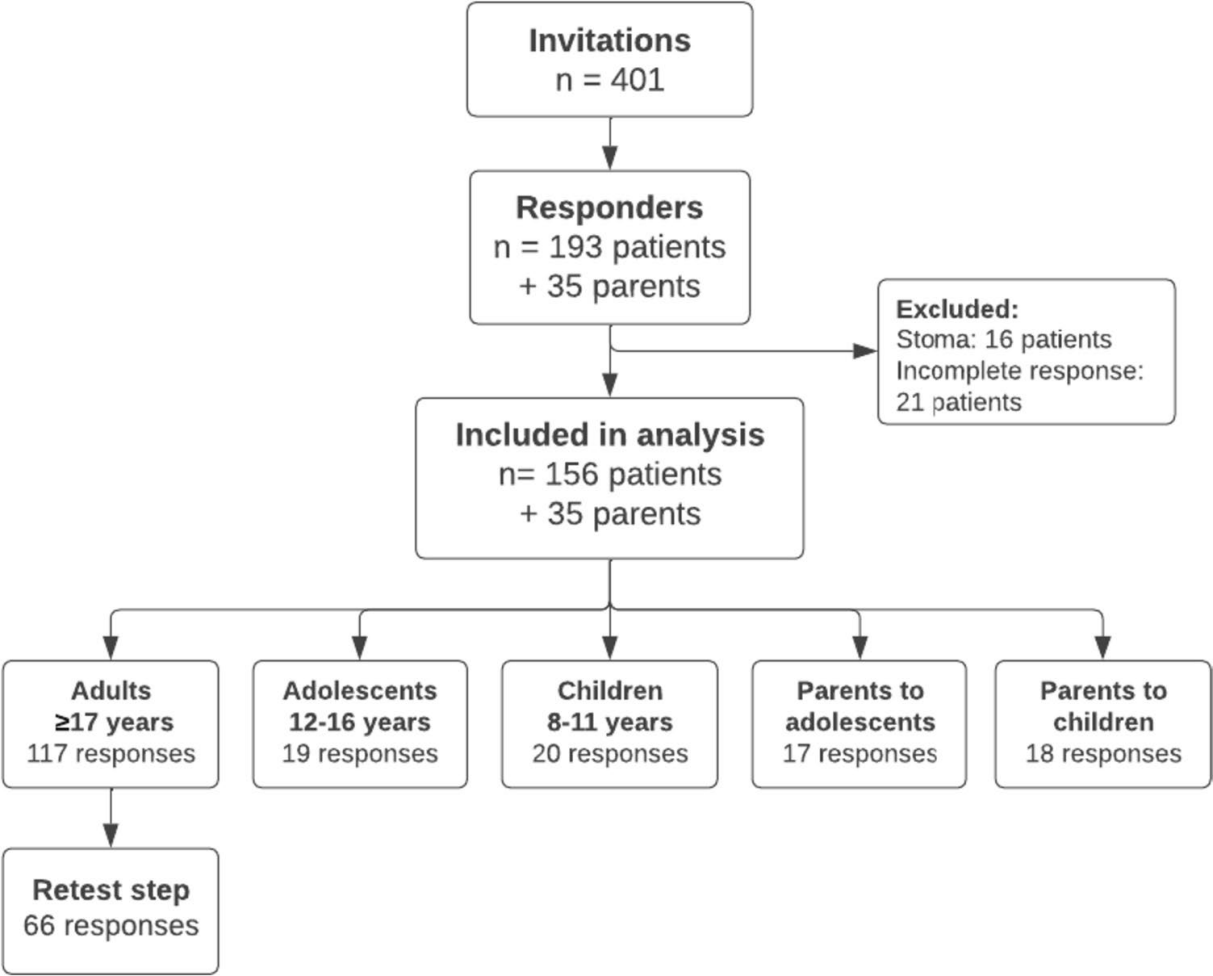
Flow diagram of included questionnaires

The number of eligible patients, responders and included responses can be found in Fig. 1. From a total of 401 eligible patients, 193 consented to participate as well as 35 parents. The response rates for each of the five different HAQL questionnaires can be found in Table 3. The response rate for the retest step for adults, who completed the questionnaire, was 52% (*n* = 66).

**Table 3:** Number of invitations and response rates for each of the HAQL questionnaires

Questionnaire	Number of invitations	Response rate, % (*n*)
HAQL_ADULT_	272	54% (148)
HAQL_12–16_	71	31% (22)
HAQL_PARENT 12–16_	71	24% (17)
HAQL_-8–11_	58	40% (23)
HAQL_PARENT 8–11_	58	31% (18)

A total of 16 responses were excluded from statistical analysis (10 adults, 3 adolescents and 3 children) due to patients’ indication of having a stoma. A total of 21 responses to questionnaires (all adults) were excluded from statistical analysis due to a less than 50% completion of the questionnaire. Thus, a total of 156 responses from patients and 35 responses from parents were available for analyses.

### Face validity

Ninety-six percent of responders did not report any difficulties in understanding the items. Six patients (one child and five adults) reported difficulties with understanding individual and different words or questions.

The question of relevance of the items was addressed with a very open-ended question, which prompted very diverse answers reflecting the very diverse nature of the disease. Most patients indicated that they found the items were relevant without any suggestions for further items. Several patients indicated a frustration that items used a short (1-week) time frame.

The rate of participants choosing the response “I do not wish to reply” for any item was 1.4% for the adults, 2.7% for the adolescents, 0% for the adolescent’s parents, 0.4% for children and 0.6% for the children’s parents. For the retest step for the adults, the rate was 2.0%. The highest response rates of “I do not wish to reply” were found within the items related to sexual functioning (up to 9%).

### Reliability and validity of the original structure

The results of the reliability and validity analysis can be found in Table 4. The internal consistency of the dimensions was overall satisfactory for adults and adolescents’ questionnaires but more problematic for the children’s questionnaire, where the Cronbach’s alpha was less than 0.7 in 60% of the dimensions.

**Table 4:** Number of patients (*N*) who answered > 50% of each dimension

HAQL dimension	*N*	% missing	Min score	Max score	Mean score	SD	Internal consistency (Cronbach’s alpha coefficient)	Intraclass correlation coefficient
Adults								
Laxative diet	117	0	0	100	87.9	22.5	0.6564	0.73
Constipating diet	117	0	16.7	100	87.9	20.6	0.4878	0.60
Presence of constipation	117	0	0	100	76.6	26.4	–	0.59
Presence of diarrhea	114	3	0	100	68.7	30.2	0.8184	0.68
Fecal incontinence	116	1	37.5	100	91.8	11.1	0.7173	0.83
Urinary incontinence	116	1	0	100	96.6	11.9	0.9029	0.72
Social functioning	115	2	0	100	93.3	14.9	0.7583	0.72
Emotional functioning	116	1	38.1	100	87.0	16.2	0.7888	0.82
Body image	116	1	0	100	77.4	29.8	0.8139	0.70
Physical symptoms	116	1	20	96.7	68.7	17.0	0.7886	0.81
Sexual functioning	106	9	0	100	91.0	19.8	0.8467	0.70
Overall scale	103	12	41.8	99.4	85.0	11.4	0.7765	0.79
Adolescents								
Laxative diet	19	0	16.7	100	93.0	20.3	0.7747	0.15
Constipating diet	19	0	33.3	100	80.7	22.4	0.2589	0.50
Presence of constipation	17	11	33.3	100	90.2	19.6		0.35
Presence of diarrhea	17	11	0.0	100	45.1	41.6	0.7569	0.85
Fecal incontinence	19	0	37.5	100	76.8	21.9	0.8932	0.78
Urinary incontinence	19	0	41.7	100	94.0	18.0	0.8668	0.00
Social functioning	18	5	53.3	100	85.9	14.6	0.4482	0.91
Emotional functioning	18	5	50.0	100	85.0	17.7	0.6750	0.81
Body image	18	5	33.3	100	77.8	24.9	0.5673	0.63
Physical symptoms	19	0	33.3	93.3	69.2	18.8	0.8072	0.45
Overall scale	15	21	54.8	98.9	80.0	14.6	0.8534	0.86
Children								
Laxative diet	20	0	0.0	100	82.5	34.4	0.9191	0.90
Constipating diet	20	0	33.3	100	93.3	16.6	0.5638	0.58
Presence of constipation	19	5	33.3	100	89.5	22.4		0.13
Presence of diarrhea	19	5	0.0	100	57.0	30.6	0.6098	0.90
Fecal incontinence	20	0	45.8	100	81.7	16.2	0.7622	0.78
Urinary incontinence	20	0	66.7	100	96.7	8.7	0.5841	0.76
Social functioning	20	0	66.7	100	89.0	10.2	0.3906	0.67
Emotional functioning	20	0	50.0	100	80.4	16.5	0.5455	0.55
Body image	20	0	0.0	100	75.8	30.8	0.6836	0.67
Physical symptoms	20	0	16.07	93.3	61.7	18.7	0.7309	0.76
Overall scale	19	5	58.8	96.8	81.1	10.9	0.7123	0.87

Scoring and reliability for each item within the three different patient questionnaires. The intraclass coefficient is reported for the test–retest step for the adults and for the patient-proxy relationship for children and adolescents

The ICC reported for the test–retest step in adults was below 0.6 for the dimensions for “presence of constipation”, only. The ICC for the patient/parent questionnaires were below 0.6 in 50% of the dimensions for the adolescent/parent and in 30% the dimensions for the child/parent questionnaire.

### Dropout analysis

We were not able to make a comparison between the severity of disease (extent of aganglionosis) in responders and non-responders because we were not allowed to perform a review of the medical records for non-responders.

For the validation of the test–retest step for the adults, patients who responded to the retest step were compared to patients, who did not respond to the retest, based on their responses for the initial test. Non-responders to the retest step had significantly higher total scores compared to the responders (87.7 vs 82.6, *p* = 0.023).

### Known groups validity

For the known groups validity analysis, 85 responses were included (68 with mild HD and 17 with severe HD). The results of the comparison of patients with mild and severe HD can be found in Table 4. The only dimension, which demonstrated a significant difference, was the dimension for the presence diarrhea where patients with severe disease had a significantly decreased dimension score compared to patients with mild disease (Table 5).

**Table 5:** Dimension scores for patients with mild and severe Hirschsprung’s disease (HD) based on the anatomic extension of disease

Dimension	Mild HD *N* = 68	Severe HD *N* = 17	Difference (95%-confidence interval)	*p*-value
Laxative diet	87.01 (68)	91.18 (17)	4.17 (− 7.91; 16.25)	0.495
Constipating diet	82.11 (68)	93.14 (17)	11.03 (− 1.16; 23.22)	0.076
Presence of constipation	74.51 (68)	80.39 (17)	5.88 (− 9.45; 21.21)	0.448
Presence of diarrhea	72.22 (66)	38.24 (17)	− 33.99 (− 49.06; -18.91)	< 0.001
Fecal incontinence	90.56 (68)	89.46 (17)	− 1.10 (− 7.65; 5.45)	0.739
Urinary incontinence	95.96 (68)	96.08 (17)	0.12 (− 7.25; 7.49)	0.974
Social functioning	91.67 (67)	93.63 (17)	1.96 (− 6.84; 10.76)	0.659
Emotional functioning	86.06 (68)	84.03 (17)	− 2.03 (− 10.95; 6.89)	0.652
Body image	75.00 (68)	69.61 (17)	− 5.39 (− 22.41; 11.63)	0.530
Physical symptoms	69.16 (68)	66.86 (17)	− 2.29 (− 11.19; 6.60)	0.610
Sexual functioning	91.11 (60)	84.38 (16)	− 6.74 (− 19.21; 5.74)	0.285
Overall scale	84.32 (58)	80.35 (16)	− 3.97 (− 10.58; 2.63)	0.234

The numbers in brackets are the number of patients, who completed > 50% of the items in each dimension

## Discussion

The present study showed an overall satisfactory content and face validity as well as reliability of the Danish translated version of the HAQL questionnaires. The translation process resulted in minor changes to the social functioning, physical functioning, and emotional functioning dimensions. These changes did not seem to affect the performance of the questionnaire as the internal consistency and intraclass correlation for these dimensions were similar to other translated versions of the questionnaires.

The response rates were relatively low in the present study with 54% for adults and lower for children/adolescents and lowest for parents. The response rates were comparable with the French study [5] but considerably lower compared to the 62% response rate in the original study from the Netherlands [4]. There is no information on the response rate from the Swedish study [7]. Response rate may depend on the approach to the patients and how many reminders that are allowed to be send [12]. In our study the patient and parent approach were pure electronic, and we cannot exclude that an approach with conventional mail or other forms might have increased the response rate. We chose the electronic approach because this is generally used in Denmark for the communication between the health care providers and patients.

Low response rates could cause concerns about whether the sample is representative of a wide range of disease severity. For most dimensions, however, the scores had great diversity and often included the very highest and lowest values. We therefore do not expect any serious selection bias for the results.

For the adult questionnaire, the dimensions on constipation, laxative diet and constipating diet were the only dimensions with less than satisfactory reliability analysis, which is similar to the reliability results of other translations. Constipation is an unspecific and not well-defined symptom, which may be difficult for the patients to recognize and report consistently [13]. The same applies to the dimensions on laxative or constipation diet. Furthermore, they are all dimensions with very few items, which makes them more susceptible to small differences in responses when investigating reliability.

This study also demonstrated less than satisfactory reliability within the dimension of social functioning in adolescents and children and with the emotional functioning dimension in children. The reason for this might be that the questionnaire for both groups cover a period in life with dramatic changes in individuals psychological and physical development such as school, education, human relations and puberty. One could speculate whether it would be more appropriate with questionnaires specially aimed for the physical and or psychological development of the child and not for an age-interval only. Furthermore, there might be differences between boys and girls. To evaluate this requires a much larger group of participants. Many patients may have learned to live with the disabilities and have learned to neglect the problems, and this may explain the relative low validity in answers between children/adolescents and their parents for some of the dimensions.

The 1-week time frame of the items was challenged and questioned by several patients in the comment-section at the end of the questionnaire. Patients commented that their symptoms varied considerably over time and that the last week might not be representative of their “mean state”. If the items had a longer or no timeframe, patients may be susceptible to memory bias [14]. The test–retest step of the trial showed satisfactory results, which illustrates that, at least for the patients who answered the retest step, symptoms were relatively stable over time.

In this study we chose to exclude patients with a terminal stoma, since this group was too small for statistical analysis. None of the other available studies have included this group of patients.

The present questionnaires may be an important instrument to measure and validate the long-term outcome after different treatment modalities or surgical procedures for HD. To evaluate this, we performed a subanalysis on the score for patients with mild HD or severe HD. Unexpectantly, we found only a significant difference in the score for diarrhea. The clinical experience is that patients with more extensive aganglionosis often have a more complicated course and a worse functional outcome. An explanation could be that the study was underpowered to demonstrate differences. We suggest that future use of the HAQL questionnaire be supplemented with generic QoL instruments as well as recording of demographic data such as education, marital status, childbirth, income and healthcare utility. to provide a more thorough evaluation of QoL in patients operated for HD.

This study has several limitations. First, it was translated form the Swedish version and not from the original Dutch version. We had to make this compromise due to the lack of sufficient linguistic competences between the Dutch and Danish language. The Swedish version has been very thoroughly translated and culturally adapted and due to the linguistic and societal similarities, we considered it most convenient and reliable to translate the Swedish version. Another limitation might be the inclusion of HD patients alone and not anorectal malformations, but this could also be a strength. The two different malformations have a lot in common but also some important differences both in etiology and surgical methods.

A limitation might be that we didn’t include a concurrent validity. This would require completion of questionnaires on generic indices of QoL. The problems with these are the difference in the subscales investigated. In the original study a low correlation between TACQOL for children was found and significant correlation was reported for a few subscales, only, in the F-36 for adults [4].

In conclusion the present format of the HAQL questionnaires have some strengths but also some weaknesses that calls for an improvement especially for children and adolescents.

In conclusion the translated version of the HAQL questionnaires provides an overall reliable instrument for evaluating disease-specific QoL in a Danish HD population, but it is important to acknowledge the limitations of the questionnaire, especially in children and adolescents.

## Data Availability

No datasets were generated or analysed during the current study.
